# Nanoemulsion of *Gomortega keule* Essential Oil: Characterization, Chemical Composition, and Anti-Yeast Activity Against *Candida* spp.

**DOI:** 10.3390/pharmaceutics17060755

**Published:** 2025-06-08

**Authors:** Iván Montenegro, Bastián Fuentes, Valentina Silva, Francisca Valdés, Enrique Werner, Rocío Santander, Daniel Moraga-Espinoza, Alejandro Madrid

**Affiliations:** 1 Center of Interdisciplinary Biomedical and Engineering Research for Health (MEDING), Escuela de Obstetricia y Puericultura, Facultad de Medicina, Universidad de Valparaíso, Angamos 655, Reñaca, Viña del Mar 2520000, Chile; ivan.montenegro@uv.cl (I.M.); bastianfuentes.valdes@gmail.com (B.F.); 2 Laboratorio de Productos Naturales y Síntesis Orgánica (LPNSO), Facultad de Ciencias Naturales y Exactas, Universidad de Playa Ancha, Leopoldo Carvallo 270, Playa Ancha, Valparaíso 2340000, Chile; silvapedrerosv@gmail.com (V.S.); fvaldesnavarro@gmail.com (F.V.); 3 Departamento de Ciencias Básicas, Campus Fernando May, Universidad del Bío-Bío, Avda. Andrés Bello 720, Casilla 447, Chillán 3780000, Chile; ewerner@ubiobio.cl; 4 Kinetics and Photochemistry Laboratory, Department of Environmental Sciences, Faculty of Chemistry and Biology, Universidad de Santiago de Chile, Santiago 9170022, Chile; rocio.santanderm@usach.cl; 5 Laboratorio de Tecnología Farmacéutica, Facultad de Farmacia, Escuela de Química y Farmacia, Universidad de Valparaíso, Valparaíso 2340000, Chile; daniel.moraga@uv.cl

**Keywords:** *Gomortega keule*, essential oil, *Candida* spp., nanoemulsion

## Abstract

**Background/Objectives:** Candidiasis, an opportunistic fungal infection caused by Candida species, is a major health problem, particularly in immunocompromised individuals. Increasing resistance of yeasts such as *Candida* spp. to pharmacological antifungal agents makes it necessary to explore alternative treatments. The aim of this study was to evaluate the antifungal potential of *Gomortega keule* essential oil (GKEO) against *Candida* spp. by assessing growth and development at 24 and 48 h. Encapsulation and characterization of a stable nanoemulsion were carried out to enhance efficacy. **Methods**: The anti-yeast activity of both free GKEO and the nanoemulsion against *Candida albicans*, *C. glabrata*, and *C. guilliermondii* was evaluated using a microdilution method to determine the minimum inhibitory concentration (MIC) and minimum fungicidal concentration (MFC) at 24 and 48 h. **Results**: GC-MS/MS analysis identified forty-one components in GKEO, the main ones being eucalyptol (21.41%), 4-terpineol (19.62%), and α-terpinyl acetate (13.89%). Antifungal assays revealed that both free and nanoemulsion-formulated GKEO inhibited the growth of all tested *Candida* strains. At 48 h, the nanoformulated GKEO achieved a MIC value of 32 µg/mL and an MFC of 64 µg/mL for *C. albicans* and *C. glabrata* and showed higher antifungal activity compared to the free oil, in particular against *C. albicans*, exhibiting a four-fold lower MFC value. The activity of the nanoformulation was comparable to or better than fluconazole against *C. glabrata* and *C. guilliermondii*. **Conclusions**: The GKEO nanoemulsion potentiated anti-yeast activity against *Candida* spp. The formulation improved the efficacy of GKEO, suggesting its potential as an alternative or adjunctive treatment for candidiasis.

## 1. Introduction

Candidiasis is an opportunistic infection due to *Candida*, a form of yeast, which can affect the oral cavity, vagina, or other parts of the body. Untreated *Candida* infection carries the risk of leading to a systemic infection in which other organs can progress to systemic infection and death [1]. This type of yeast pathology occurs most frequently as a secondary infection in immunocompromised individuals [2]. The most frequent forms worldwide are oral and vaginal candidiasis, characterized by an acute inflammation of the corresponding mucosa due to overgrowth of normally commensal *Candida* species [3,4]. Although *Candida albicans* is the predominant cause of Candidiasis, the prevalence of species like *C. tropicalis*, *C. parapsilosis*, *C. glabrata*, *C. krusei*, *C. guilliermondii*, *C. dubliniensis*, and *C. auris* has increased considerably in recent years [5]. Candida infections are treated with antifungals like nystatin, clotrimazole, amphotericin B, and miconazole. For mild genital infections, antifungal creams (1- to 7-day treatments) or a single oral dose of econazole or fluconazole can be used [6]. However, increasing resistance to these antifungals among *Candida* species is impacting clinical effectiveness [7]. Overuse of inappropriate antifungals, systemic toxicity, drug interactions, and the limited number of effective antifungals have contributed to this growing resistance and its clinical limitations [8]. Due to the reduced effectiveness of many antifungals, there’s a pressing need for alternative treatments for conditions like oral and vaginal candidiasis. Many natural substances, including essential oils, possess antifungal properties and alternative treatments. A number of studies have shown that essential oils inhibit the growth of microorganisms by increasing cell membrane permeability, disrupting the cell membrane, causing leakage of vital intracellular components and interfering with cell metabolism and enzyme kinetics [9]. This antimicrobial capacity, together with antioxidant, anticancer and anti-inflammatory actions, has made essential oils valuable agents for the pharmaceutical, cosmetic, and food industries [10].

The Gomortegaceae family contains a single genus, *Gomortega*, with only one species, *Gomortega keule* (Mol.) Baillon, common names “queule” or “hualhual”, which is endemic to the coastal mountain range of south-central Chile [11]. Essential oil of *G. keule* can be found in its leaves and has shown insecticidal and anti-phytopathogenic activities [12,13].

Despite their biological properties, essential oils face limitations in application due to poor stability and water solubility. Formulating essential oils as nanoemulsions offers a solution by improving both stability and solubility. Nanoemulsions also facilitate lymphatic transport, enhance mucosal permeability, and increase the bioavailability of medicinal components [14].

Therefore, this study explored the anti-yeast activity of *G. keule* essential oil (GKEO) by utilizing a characterized nanoemulsion as a delivery system. The goal was to develop a stable oil carrier system with the potential to combat various *Candida* strains.

## 2. Materials and Methods

### 2.1. Plant Material

Leaves of *G. keule* were collected in April 2024 from the “Reserva Nacional Los Queules”, Pelluhue, Maule Region, Chile. A voucher specimen is kept at the Laboratorio de Productos Naturales y Síntesis Orgánica (LPNSO) of the Universidad de Playa Ancha, Valparaíso, Chile (GK-04-24), and its identity was confirmed by the botanical expert Patricio Novoa.

### 2.2. Essential Oil

The essential oil of *G. keule* was obtained by hydrodistillation of 300 g of fresh leaves using a Clevenger-type apparatus for 4 h, under controlled temperature conditions, at approximately 100 °C. The oil obtained was dried over anhydrous sodium sulfate and stored in a sealed amber bottle at 4 °C for further analysis.

### 2.3. Characterization of Essential Oil

The GKEO was diluted with dichloromethane, and 1 μL of the sample was analyzed using a GC-MS/MS (GC: model Trace 1300 and MS: model TSQ8000Evo, Thermo Fisher Scientific, Waltham, MA, USA) operating in EI mode at 70 eV, equipped with a splitless injector (250 °C). The transfer line temperature was 200 °C. Helium was used as a carrier gas at a flow rate of 1.2 mL/min, and the capillary column used was an Rtx-5 ms (60 m × 0.25 mm i.d., film thickness 0.25 μm). The temperature program was 40 °C (5 min) to 300 °C (5 min) at a rate of 5 °C/min. The chemical composition of the oil was identified by comparing its spectra with an NIST20 library (using a match value > 800 as the acceptance criterion) [15] and confirmed by comparing the retention indices with data published in other studies.

### 2.4. Nanoemulsions

#### 2.4.1. Preparation of the Nanoemulsion

The nanoemulsion was prepared following the procedure proposed by Maruno et al. [16], with modifications. This modified procedure involved heating the oil and aqueous phases separately to 75 ± 2 °C. First, the oil phase (olive oil and octanol) was added to the aqueous phase (distilled water, Pluronic F127, and Tween 80). This mixture was immediately agitated at 21,500 rpm for five minutes using an Ultra-Turrax (IKA, model T25 basic, Staufen, Germany). Subsequently, the sample was placed in an ice bath and mechanically stirred at 500 rpm for 2 min. GKEO was then slowly added while stirring continued for another 2 min. Finally, after homogenization, the resulting emulsion was subjected to an ultrasonic bath for 10 min to ensure complete dispersion of the oil phase within the aqueous medium. The formulation was stored at room temperature for 7 days and subsequently characterized and evaluated for its stability prior to use. The nanoemulsion was formulated using the following concentrations (% *w*/*v*, weight/volume): 1% GKEO, 1% olive oil, 2% octanol, 5% Tween 80, and 5% Pluronic F127.

#### 2.4.2. Determination of Particle Size and Zeta Potential

The mean particle size and zeta potential of the nanoemulsions were measured using dynamic light scattering (DLS) and phase analysis light scattering (PALS), respectively, with a Zetasizer Nano ZS system (Malvern Instruments, Malvern, UK). The formulations were diluted 200-fold with distilled water prior to measurement to ensure the accuracy of the results.

#### 2.4.3. Encapsulation Efficiency (EE)

The encapsulation efficiency (EE) of GKEO was determined by gas chromatography–mass spectrometry (GC-MS) and calculated using Equation (1), according to the previously described method [17].

EE (%) = (GKEO encapsulated in nanoemulsion/total GKEO added) × 100,(1)

#### 2.4.4. Morphological Analysis

Scanning electron microscopy (SEM) analysis was conducted using a ThermoFisher FE-STEM equipped with an FEI Quanta FEG 250 (Waltham, MA, USA). The 3+ detector facilitated high-resolution imaging, revealing the spherical morphology and uniform size distribution of the nanoemulsion droplets. SEM was employed to complement dynamic light-scattering (DLS) measurements, ensuring that the observed particle sizes were not artifacts of agglomeration, aligning with best practices in nanoparticle characterization.

### 2.5. Biological Assay

#### 2.5.1. Strains

The GKEO and its nanoformulation were tested against three clinical strains of *Candida* spp: *C. albicans* 10935 (isolated from a pulmonary infection), *C. glabrata* 10912, and *C. guilliermondii* 12204 (both isolated from urinary tract infections). The strains were obtained from patients of the Base Hospital of Valdivia, Los Ríos Region, Chile. After identification, the microorganisms were included in the pathogenic fungal collection (Bioassay Laboratory of University of Valparaíso). They were maintained in Sabouraud Dextrose Broth (SDB) with glycerol at −80 °C according to established protocols [18].

#### 2.5.2. Anti-Yeast Assay

The minimum inhibitory concentration (MIC) was determined by the microdilution method for yeast, with slight modifications [19,20]. Briefly, cultures of all yeast strains were placed on Sabouraud dextrose agar (SDA) and incubated for 24–72 h at a temperature of 37 °C. Colonies of this culture were suspended in sterile 0.85% NaCl, and the inoculum was standardized according to the scale of 0.5 McFarland (1–5 × 10^6^ CFU/mL). The antifungal test was performed in 96-well plate. Yeast strains were prepared in sterile water and diluted in RPMI 1640 medium (except in the sterility control). Essential oil and compounds were dissolved in dimethyl sulfoxide (DMSO) at final concentrations of 256 to 0.03 µg/mL. The MIC_80_ determination was conducted with approximately 0.5–2.5 × 10^3^ CFU/mL of the microorganism in each well. The plates were incubated at 37 °C for 24–48 h and absorbance was measured at 540 nm [21]. The MIC endpoint was defined as the lowest concentration that resulted in ≥80% growth inhibition compared to the growth control (MIC_80_), following the visual endpoint criterion recommended by the NCCLS.

After determining the MIC, the minimum fungicidal concentration (MFC) was determined by subculturing 2 μL from each well that showed no visible growth. The subcultures were incubated at 35 °C for 72 h. The MFC was defined as the lowest concentration with no visible growth, indicating a 99.5% reduction of the original inoculum [19]. Fluconazole, voriconazole, and itraconazole were used as positive controls. All experiments were performed in triplicate and repeated three times to ensure reproducibility.

## 3. Results and Discussion

### 3.1. Oil Composition

The essential oil was obtained from plant material with a yield of 0.99% (*v*/*w*). The qualitative and quantitative composition of the oil is shown in Table 1. Forty-one components representing 95.57% of the volatile oil were identified. The oil was characterized by high amounts of eucalyptol (21.41%), 4-terpineol (19.62%), and α-terpinyl acetate (13.89%), and to a lesser extent by the sesquiterpenes α-calacorene (6.49%) and δ-cadinene (6.49%) and the oxygenated monoterpenes α-terpineol (4.27%) and myrtenal (2.38%).

This essential oil was previously characterized with a high percentage of 1.8 cineol (35.57%) and a high percentage hydrocarbon monoterpene such as α-pinene (7.30%), α-terpinene (7.17%), limonene (5.40%), β-pinene (5.30%) and 3-carene (5.17%) [12]. In contrast to our results, although the percentage abundance of the major compound eucalyptol or 1,8 cineol is similar, the presence of monoterpenes differs completely since the oil obtained by us was characterized by a high concentration of oxygenated terpenes. In another case, only five compounds are reported to be present in GKEO where aromatic compounds such as naphthalene, azulene and anthracene predominate with 57.0%, 5.3%, and 4.5% of abundance, respectively, added to the diterpene kaurene with 20.7% of abundance [13]. These results differ significantly from ours, both in terms of the number of compounds identified and their relative abundance.

### 3.2. Nanoemulsion Formulation

Utilizing 5 min of ultrasonic cavitation, the GKEO nanoemulsion was formulated with a mixed surfactant system of Pluronic F127 (poloxamer) and Tween 80 in the aqueous phase, and a dispersed oily phase of vegetable oil and octanol. The component selection emphasized safety and regulatory compliance. The surfactants are non-toxic, while octanol, an alcohol approved by the Food and Drug Administration (FDA) and Environmental Protection Agency (EPA) for use as a food flavoring [22,23], exhibits a favorable toxicological and environmental profile. Crucially, it is not genotoxic, displays no repeated dose toxicity, and doesn’t negatively impact fertility or development (margin of exposure, MOE > 100). Further, the risk of skin sensitization (no expected sensitization induction level, NESIL of 10,000 μg/cm^2^) and photoirritation/photoallergy is minimal, and local respiratory exposure remains below levels of concern. Environmentally, it is classified as non-persistent, non-bioaccumulative, and non-toxic, with low risk quotients (predicted environmental concentration/predicted no effect concentration, PEC/PNEC < 1), based on data from octanol and its analogs, heptyl alcohol and 1-decanol [24]. Adding to its appeal, octanol has shown promise as a therapeutic agent: a single oral dose of 1 mg/kg resulted in a significant reduction in tremor amplitude in a randomized, placebo-controlled trial involving 12 patients with essential tremor, without eliciting significant side effects or intoxication [25].

Immediately after manufacture (Day 0) the formulation displayed a mean hydrodynamic diameter of 22 nm. In accordance with the long-term protocol specified by ICH Q1A(R2), the same batch was re-analyzed after 210 days (≈7 months, i.e., beyond the required six-month checkpoint). The mean size remained 22 nm, with no statistically significant difference from Day 0, indicating sustained physical stability over this interval. While these results are encouraging, the 12-month time point has not yet been reached, so a definitive shelf-life claim cannot be made. Our process builds on the approach of Azevedo et al. (2015), where a nanoemulsion was generated together with *Opuntia ficus-indica* extracts as a wetting agent [26]. We used this methodology as a foundation, applying the necessary modifications to adapt it to our formulation, as this type of nanoemulsion is suitable for topical application. This is relevant to our research because *Candida* species infect mucosal epithelia and can also cause superficial mycoses [27].

### 3.3. Nanoemulsion Characterization

The characterization of the nanoemulsion with and without GKEO is shown in Table 2.

The determination of particle size, polydispersity index, pH, and zeta potential are some of the parameters most used to evaluate the stability of nanoemulsions, since particle size interferes with flocculation and coalescence phenomena [28].

The GKEO nanoemulsion exhibited optimal particle sizes, falling within the desired 20–200 nm range for nanoemulsions. This size range promotes good stability and minimizes issues such as precipitation or sedimentation [29].

The PDI, with values ranging from 0.0 to 1.0, serves as an indicator of particle distribution uniformity. A PDI closer to 0 signifies a more uniform distribution of particles. In the case of the GKEO, the PDI values were consistently below 1, indicative of a homogeneous droplet size distribution across all formulation groups [30].

The nanoemulsion’s pH was near 7, which is ideal for topical or cutaneous administration, as maintaining a pH between 4 and 8 is essential. A nanoemulsion pH below 4 can cause skin irritation, while a pH above 8 can lead to dry skin [31].

The zeta potential value of −4.27 mV obtained for the GKEO nanoelmulsion demonstrates a good level of stability in the emulsion system, with no evidence of flocculation. In particular, zeta potential values higher than ±30 mV indicate moderate stability in the colloidal system, which means the absence of flocculation or aggregate formation, and indicates a high stability [30].

An 82.51% encapsulation efficiency (EE) of GKO was obtained using essential oil encapsulated with Tween 80 and Pluronic F127. This study indicates the influence of Tween 80 and Pluronic F127 as stabilizers. Finally, the uniformity of particle size indicated by the low polydispersity of these dispersions demonstrates the efficacy of both surfactants as a stabilizer and a promising result for future in vivo applications.

### 3.4. Morphology

**Figure 1:**
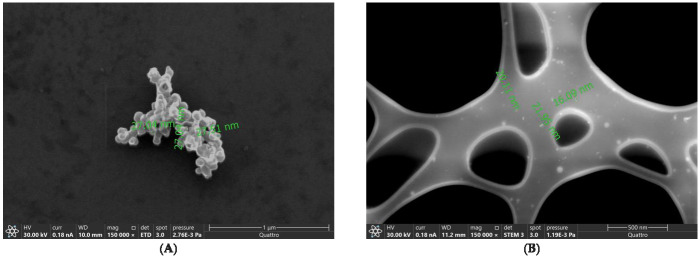
(**A**) SEM image of nanoemulsion formulation, (**B**) STEM image of nanoemulsion.

SEM and STEM images of GKEO nanoemulsion revealed homogeneous and regular shaped droplets with clear contours and cores (Figure 1), well segmented from the background.

The average particle size determined by dynamic light scattering (DLS) was 22 nm (Supplementary Data), aligning well with measurements from scanning electron microscopy (SEM), which showed nanoemulsion droplets ranging from 27 to 28 nm, and scanning transmission electron microscopy (STEM), which indicated sizes between 16 and 22 nm (Figure 1). This concordance validates the DLS results, confirming that the measured sizes represent individual particles rather than agglomerates. Employing advanced microscopy techniques alongside DLS is recommended to overcome DLS’s limitations in distinguishing between single particles and aggregates, as acknowledged in regulatory standards such as ASTM E3247-20 and FDA guidance documents (ASTM E3247-20, FDA Guidance) [32,33].

### 3.5. Antifungal Activity

The minimum inhibitory concentrations (MICs) of *G. keule* essential oil, both in its free form and loaded in a nanoformulation at 24 and 48 h, are shown in Table 3 and Table 4, respectively. Table 3 also presents the minimum fungicidal concentration (MFC) for both samples.

Antifungal evaluation revealed that the essential oil exhibited activity against all tested *Candida* strains. Table 2 shows that GKEO displayed 24 h MIC values ranging from 0.5 to 32 µg/mL against these strains. Specifically, the free oil exhibited a potent antifungal activity with an MIC of 0.5 µg/mL at 24 h. This was 16-fold and 2-fold more potent than its nanoformulation against *C. guilliermondii* and *C. glabrata*, respectively. Conversely, against *C. albicans*, the free oil was two-fold less active than the nanoformulation. Compared to the positive controls, the free oil demonstrated a strong antifungal effect, particularly against *C. guilliermondii*, exceeding the activity of fluconazole and voriconazole and matching that of itraconazole. Against *C. glabrata*, the MIC of the free GKEO was four-fold lower than fluconazole, comparable to voriconazole, and eight-fold higher than itraconazole. However, the activity of the free EO against *C. albicans* was low compared to the control treatments.

Compared to 24 h results, the 48 h MIC and MFC data (Table 3) indicate a reduction in the activity of the free GKEO, while the nanoformulated GKEO exhibited enhanced antifungal activity. Both the free EO and the nanoemulsion remained effective against all tested strains. In this context, the nanoformulation’s activity was significantly more potent than the free oil, particularly against *C. albicans*, exhibiting a 4-fold lower MFC value. Against *C. glabrata*, the MFC value was two-fold lower, and against *C. guilliermondii*, the MFC values were identical. When compared with pharmaceutical controls, the nanoformulation demonstrated superior effectiveness to fluconazole, with a two-fold lower MFC value against both *C. guilliermondii* and *C. glabrata*. However, it showed lower activity against *C. albicans*. The nanoformulation’s effectiveness was comparable to voriconazole against *C. glabrata* and *C. guilliermondii*, but significantly lower against *C. albicans*. Finally, itraconazole was more effective than the nanoformulation against both *C. glabrata* and *C. guilliermondii* but showed comparable effectiveness against *C. albicans*.

The MFC/MIC ratio describes treatment effect. A high ratio indicates tolerance. Conversely, it has been established that ratios of 1 or 2 suggest that a fungicidal effect has occurred. When ratios equal to or greater than 4 are achieved, it is considered fungistatic [34]. As shown in Table 3, treatments with GKEO and its nanoformulation maintained a ratio of 2, highlighting that the nanoformulation achieved a ratio of 1 in *C. guilliermondii*. Among all samples tested it can also be noted that two of the positive controls have a ratio of 4; voriconazole in *C. glabrata* and itraconazole for *C. guilliermondii*.

The activity of essential oils, being complex mixtures, is difficult to attribute to a single component. However, it is plausible that the activity of this oil is largely due to the presence of high concentrations of eucalyptol (21.41%), 4-terpineol (19.62%), and α-terpinyl acetate (13.89%). Eucalyptol, a natural compound found in various plants, including eucalyptus, rosemary, and camphor, has demonstrated a potent antifungal effect against a wide range of *Candida* species, including strains of *C. albicans* (ATCC 10231, 475/15, 527/14, 10/15, 27/15, 532/15, 503/15, 13/15, 16/15), *C. tropicalis* (ATCC 750), *C. parapsilosis* (ATCC 22019), *C. krusei* (H1/16), and *C. glabrata* (4/6/15) [35]. Studies have shown that eucalyptol effectively inhibits fungal growth and biofilm formation, with minimum inhibitory concentrations (MICs) ranging from 2 to 23 mg/mL. This remarkable antifungal potential reinforces the value of eucalyptol, in addition to its well-documented anti-inflammatory, antioxidant, and antimicrobial properties [36].

Besides eucalyptol, the antifungal activity of GKEO could be attributed to the presence of terpinen-4-ol, a bioactive component also found in tea tree oil [37]. This compound exhibits antifungal activity against *C. albicans*, even in azole-resistant strains, as demonstrated in in vitro studies where it inhibited growth and showed a fungicidal effect. In an in vivo rat vaginal infection model, terpinen-4-ol accelerated the elimination of *C. albicans*, including resistant strains [38]. Furthermore, the combination of terpinen-4-ol with nystatin demonstrated synergistic antifungal activity against biofilms of *C. albicans* and *C. tropicalis*, inhibiting growth and reducing the adhesion of *C. tropicalis* to oral cells. The combination of terpinen-4-ol and nystatin emerges as a promising alternative against fungal infections, due to its enhanced activity and its ability to inhibit adhesion [39]. While eucalyptol and terpinen-4-ol show promising antifungal activity, α-terpinyl acetate shows mixed results. A study on *Thymus pulegioides* essential oil chemotype α-terpinyl acetate (α-TA) and pure α-TA revealed relatively weak activity against *C. albicans* and *C. parapsilosis*, with MIC of 8.00 µg/mL, higher than those of itraconazole. Overall, *Candida* yeasts proved more resistant to α-TA than other fungi and dermatophytes, with only *C. parapsilosis* showing slightly higher sensitivity than *C. albicans*. This underscores the variability in antifungal efficacy among different compounds and the importance of selectively investigating those with the greatest potential [40]. The efficacy is likely due to the synergy among the oxygenated monoterpenes in the essential oil. The major components could play a primary role, which is further enhanced by the presence of other compounds like α-terpineol (4.27%) and myrtenal (2.38%). Both components have shown an effect on *Candida* species [41,42].

In light of the significant antibacterial and antifungal activity of oxygenated monoterpenes [43,44], GKEO presents a potential resource for controlling or treating candidiasis while also promoting the conservation of this natural monument as a non-timber forest resource. This oil, rich in antifungal monoterpenes and possessing an appealing color and scent, could serve as an additive or active ingredient in cosmetic or pharmaceutical products. The fact that nanoformulation has shown improved results compared to the oil in its free state in vitro further supports this development. However, the volatility, susceptibility to degradation, and poor water solubility of essential oils can limit their therapeutic application. Nanoformulation addresses these limitations by protecting the essential oil from environmental degradation and enabling a controlled release of the active compounds, resulting in enhanced antifungal activity compared to free essential oils [45,46]. Future studies will evaluate cytotoxicity, anti-inflammatory, and antimicrobial activities of the free oil and new nanoformulations in vitro and in vivo [47,48].

## 4. Conclusions

Expectations and scientific reports supported by antifungal assays are driving the growth of the phytopharmaceuticals industry, especially when combined with nanotechnology applied to natural products, promoting new product developments. Our study aims to contribute to the moisturizing cosmetics sector by developing a reliable nanoemulsified delivery system. In this way, cosmetic products could outperform those developed using traditional methods and conventional macroemulsions. Furthermore, this research aligns with current trends in the cosmetics market, which focuses on products with plant-based ingredients. We also sought to add value to Chile’s endemic trees by incorporating a regional product, an oil-in-water (O/W) nanoemulsion with 1% GKEO, into our cosmetics.

## Figures and Tables

**Table 1:** Compounds detected in *G. keule* essential oil.

	Compound Name	Cas No.	RT	RI ^a^	Area %	Identification
1	Sabinene	3387-41-5	17.89	978.83	0.12	RI ^b^, MS
2	β-pinene	18172-67-3	18.00	981.69	0.21	RI ^b^, MS
3	α-terpinolene	99-86-5	19.47	1023.93	0.11	RI ^b^, MS
4	*p*-cymene	99-87-6	19.66	1029.84	1.83	RI ^b^, MS
5	Eucalyptol	470-82-6	19.89	1036.93	21.41	RI ^b^, MS
6	γ-terpinene	99-85-4	20.86	1065.93	0.21	RI ^b^, MS
7	Unknown	-	21.30	1078.65	0.30	-
8	Isoterpinolene	586-63-0	21.85	1094.17	0.37	RI ^b^, MS
9	Linalool	78-70-6	22.10	1101.38	1.51	RI ^b^, MS
10	Thujone	546-80-5	22.42	1112.35	0.50	RI ^b^, MS
11	*trans*-2-menthenol	29803-81-4	22.96	1130.50	0.14	RI ^b^, MS
12	Camphenol	3570-04-5	23.09	1134.81	0.10	RI ^b^, MS
13	Unknown	-	23.13	1136.13	0.10	-
14	L-*trans*-pinocarveol	547-61-5	23.55	1149.86	2.38	RI ^b^, MS
15	α-phellandrene-8-ol	1686-20-0	23.85	1159.51	0.14	RI ^b^, MS
16	Unknown	-	24.06	1166.20	0.13	-
17	Sabina ketone	513-20-2	24.13	1168.42	0.33	RI ^b^, MS
18	Pinocarvone	30460-92-5	24.28	1173.14	0.93	RI ^b^, MS
19	Unknown	-	24.41	1177.22	0.25	-
20	4-terpineol	20126-76-5	24.69	1185.92	19.62	RI ^b^, MS
21	Unknown	-	24.94	1193.60	0.29	-
22	α-terpineol	98-55-5	25.09	1198.18	4.27	RI ^b^, MS
23	Myrtenal	564-94-3	25.32	1206.19	2.38	RI ^b^, MS
24	Berbenone	80-57-9	25.73	1220.96	0.54	RI ^b^, MS
25	(*Z*)-Carveol	1197-06-4	26.09	1233.73	0.12	RI ^b^, MS
26	Carvone	99-49-0	26.80	1258.42	0.29	RI ^b^, MS
27	Unknown	-	27.03	1266.27	0.15	-
28	Bornyl acetate	76-49-3	27.84	1293.42	1.31	RI ^b^, MS
29	δ-terpinyl acetate	93836-50-1	28.67	1324.17	0.33	RI ^b^, MS
30	Hydroxycineyl acetate	57709-95-2	29.35	1349.67	1.35	RI ^b^, MS
31	α-terpinyl acetate	80-26-2	29.54	1356.69	13.89	RI ^b^, MS
32	β-elemene	515-13-9	30.81	1402.88	0.11	RI ^b^, MS
33	Caryophyllene	87-44-5	31.69	1438.48	0.21	RI ^b^, MS
34	Unknown	-	32.21	1459.06	0.12	-
35	Selina-5,11-diene	52026-55-8	32.28	1461.81	0.25	RI ^b^, MS
36	Ishwaran	26620-70-2	32.93	1487.01	1.61	RI ^b^, MS
37	Aristolochene	26620-71-3	33.34	1503.01	0.21	RI ^b^, MS
38	Unknown	-	33.42	1506.43	0.26	-
39	Unknown	-	33.60	1514.11	0.71	-
40	δ-cadinene	483-76-1	34.16	1537.75	3.40	RI ^b^, MS
41	α-calacorene	21391-99-1	34.74	1561.82	6.49	RI ^b^, MS
42	β-calacorene	50277-34-4	35.25	1582.66	0.80	RI ^b^, MS
43	Unknown	-	35.47	1591.56	0.11	-
44	Spathulenol	6750-60-3	35.67	1599.60	0.58	RI ^b^, MS
45	(-)-globulol	489-41-8	35.84	1607.25	0.34	RI ^b^, MS
46	Unknown	-	36.05	1616.73	0.22	-
47	α-corocalene	20129-39-9	36.59	1640.83	1.11	RI ^b^, MS
48	Di-epi-1,10-cubenol	73365-77-2	36.79	1649.67	0.24	RI ^b^, MS
49	Unknown	-	37.04	1660.65	0.16	-
50	Epicubenol	19912-67-5	37.12	1664.15	0.32	RI ^b^, MS
51	Unknown	-	37.39	1675.90	0.11	-
52	Cadalene	483-78-3	37.87	1696.58	2.19	RI ^b^, MS
53	Unknown	-	38.22	1712.86	0.10	-
54	Unknown	-	38.63	1732.23	0.28	-
55	Unknown	-	43.32	1960.24	0.72	-
56	Pimaradiene	1686-56-2	43.78	1983.48	0.19	RI ^b^, MS
57	Kaur-16-ene	562-28-7	45.28	2059.83	3.15	RI ^b^, MS
58	Unknown	-	45.75	2086.87	0.42	-

^a^ Experimental retention index for non-polar column; ^b^: bibliographic retention index for non-polar column, MS: mass spectra.

**Table 2:** Results of characterization of the nanoemulsion with and without GKEO.

Sample	Particle Size (nm)	PDI	PH	ZP (mV)	EE (%)
Nanoformulated GKEO	22.00 ± 7.3	0.484 ± 0.19	6.92	−4.27 ± 0.3	82.51
Nanoformulated without GKEO	23.80 ± 7.4	0.476 ± 0.22	6.89	−4.56 ± 0.4	0

PDI: polydispersity index; ZP: zeta potential; EE: encapsulation efficiency.

**Table 3:** MIC of *G. keule* essential oil (µg/mL) at 24 h, comparing free and nanoformulated forms.

Sample	Strain		
	*C. albicans*	*C. glabrata*	*C. guilliermondii*
GKEO	32	8	0.5
Nanoformulated GKEO	16	16	8
Fluconazole	2	32	16
Voriconazole	0.03	4	8
Itraconazole	4	1	0.5
DMSO	I	I	I
Tween 80	I	I	I
Pluronic F127	I	I	I

Each value represents the mean of three experiments (*p* < 0.05), performed in quadruplicate. I: inactive.

**Table 4:** MIC, MFC (µg/mL), and MFC/MIC ratios of *G. keule* essential oil at 48 h, comparing free and nanoformulated forms.

Sample	Strain								
	*C. albicans*			*C. glabrata*			*C. guilliermondii*		
	MIC	MFC	MFC/MIC	MIC	MFC	MFC/MIC	MIC	MFC	MFC/MIC
GKEO	128	256	2	64	128	2	4	16	2
Nanoformulated GKEO	32	64	2	32	64	2	16	16	1
Nanoformulated without GKEO	I			I			I		
Fluconazole	4	8	2	64	128	2	16	32	2
Voriconazole	0.125	0.125	1	16	64	4	16	16	1
Itraconazole	16	32	2	2	4	2	1	4	4
DMSO	I			I			I		
Tween 80	I			I			I		
Pluronic F127	I			I			I		

Each value represents the mean of three experiments (*p* < 0.05), performed in quadruplicate. I: inactive.

## Data Availability

All data are contained within the article and available for the scientific community.

## Supplementary Materials

The following supporting information can be downloaded at: https://www.mdpi.com/article/10.3390/pharmaceutics17060755/s1, Size Distribution Report by Intensity.
