# Supplementary material for: Nanoemulsion of Gomortega keule Essential Oil: Characterization, Chemical Composition, and Anti-Yeast Activity Against Candida spp
Source: Pharmaceutics. 2025 Jun 8;17(6):755. doi: 10.3390/pharmaceutics17060755 (PMC12196199; doi:10.3390/pharmaceutics17060755)

Size Distribution Report by Intensity

v2.2

Malvern

Sample Details

Sample Name: Met. N80 5°C 1

SOP Name: MEDICION NANO.sop

General Notes:

**File Name:**	MEDICION NANO.dts	**Dispersant Name:**	Water
**Record Number:**	23	**Dispersant RI:**	1,330
**Material RI:**	1,45	**Viscosity (cP):**	0,8872
**Material Absorbtion:**	0,001	**Measurement Date and Time:**	miércoles, 2 de abril de 2025 ...

System

**Temperature (°C):**	25,0	**Duration Used (s):**	80
**Count Rate (kcps):**	154,6	**Measurement Position (mm):**	4,65
**Cell Description:**	Disposable sizing cuvette	**Attenuator:**	9

Results

			Size (d.nm):	% Intensity:	St Dev (d.n...
**Z-Average (d.nm):**	22,00	**Peak 1:**	21,29	80,7	13,00
**PdI:**	0,484	**Peak 2:**	2474	19,3	1400
**Intercept:**	0,122	**Peak 3:**	0,000	0,0	0,000
**Result quality :**	Good				

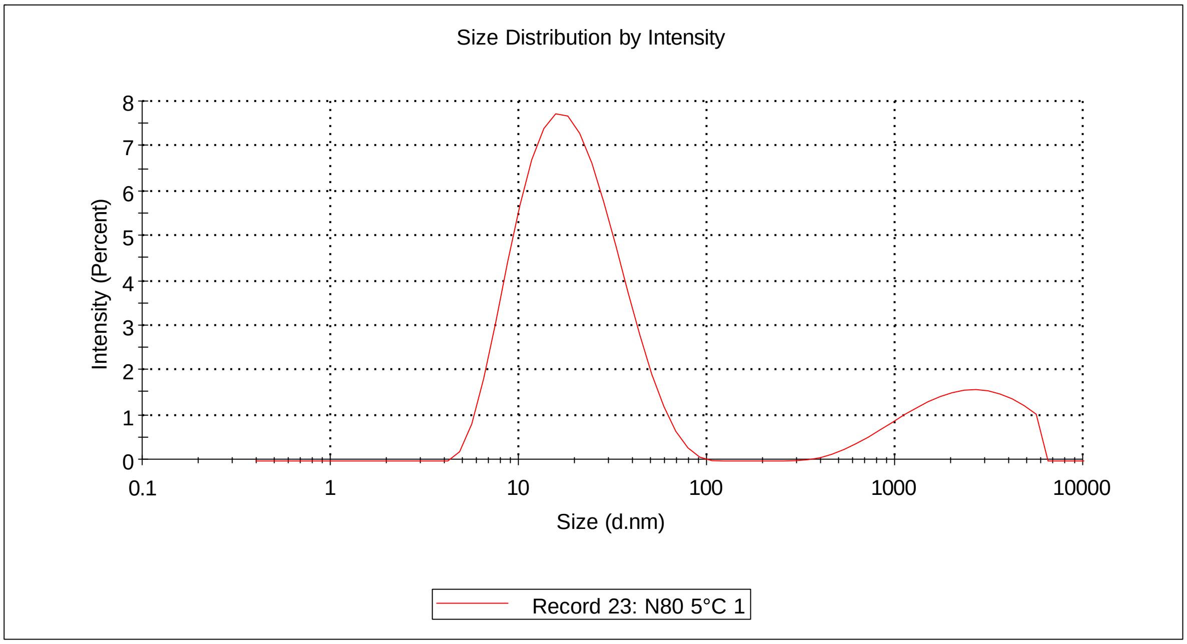

Supplement: Supplementary file 1 [file pharmaceutics-17-00755-s001.zip › pharmaceutics-3598422-supplementary.pdf]
